# Elbow medial approach open reduction and internal fixation with absorbable cannulated screws for the treatment of Kilfoyle II and III type medial condyle fractures of the humerus in children

**DOI:** 10.3389/fsurg.2025.1538399

**Published:** 2025-03-18

**Authors:** Liuyang Li, Weiqiang Li, Hong Ma, Yugeng Zheng, Yongge Chen, Huarong Ke, Yueming Guo

**Affiliations:** ^1^ Guangzhou University of Traditional Chinese Medicine, The Eighth Clinical Medical College Affiliated Guangzhou University of Traditional Chinese Medicine, Guangdong, China; ^2^ Department of Pediatric Orthopedics, Foshan Hospital of Traditional Chinese Medicine, Guangdong, China

**Keywords:** pediatric medial condyle fractures of the humerus, absorbable cannulated screws, internal fixation, elbow medial approach, children

## Abstract

**Objective:**

This study aims to investigate the clinical efficacy of elbow medial approach open reduction and internal fixation with absorbable cannulated screws for the treatment of Kilfoyle II and III type medial condyle fractures of the humerus in children.

**Methods:**

A retrospective analysis was conducted on 23 pediatric patients with medial condyle fractures of the humerus who underwent surgical treatment at the Department of Pediatric Orthopedics, Foshan Traditional Chinese Medicine Hospital, from June 2018 to December 2023. Among the patients, 15 were male and 8 were female, with ages ranging from 5 to 12 years (mean age: 9.0 ± 2.4 years). According to the Kilfoyle classification, there were 3 cases of type II fractures and 20 cases of type III fractures. Of these, 19 were fresh fractures and 4 were neglected fractures. The surgical treatment involved open reduction and internal fixation through an elbow medial approach using absorbable cannulated screws. Postoperatively, the elbow joint was fixed in a functional position using a plaster cast. Four weeks postoperatively, follow-up radiographic examination showed continuous callus formation across the fracture line. The plaster cast was then removed, and active flexion and extension exercises of the elbow joint were initiated. The final follow-up assessment was performed using the Broberg-Morrey elbow joint functional scoring system to evaluate treatment efficacy.

**Results:**

All patients were followed up for 3–12 months. All fractures achieved bony union without any breakage of the fixation devices. Among them, 2 cases had good functional scores for the elbow joint, while the others had excellent scores. None of the patients experienced complications such as infection, vascular or nerve injury, nonunion or malunion of fractures, heterotopic ossification, avascular necrosis of the medial epicondyle, or varus/valgus deformity of the elbow joint.

**Conclusion:**

Open reduction and internal fixation through an elbow medial approach using absorbable cannulated screws demonstrates favorable clinical efficacy in treating Kilfoyle II and III type medial condyle fractures of the humerus in children. It can avoid the harm of secondary surgery to children, alleviate their pain, improve elbow joint function, and is considered the preferred method for treating pediatric medial condyle fractures of the humerus, deserving promotion and application in clinical practice.

## Introduction

Pediatric medial condyle fractures of the humerus are uncommon intra-articular fractures of the elbow joint, typically occurring in children aged 8–12 years old. These fractures are even more rare in younger children and may involve injury to the trochlea of the humerus, the medial epicondyle, and portions of the humeral metaphysis, accounting for approximately 1%–2% of all elbow fractures in children (1, 2). Kilfoyle classified these fractures into three types based on the degree of displacement: Type I involves minimal displacement with an intact articular surface; Type II involves displacement of more than 2 mm without rotation; Type III involves displacement of more than 2 mm with distal and lateral rotation (3). The pediatric distal humerus has four secondary ossification centers, which appear at different ages, leading to a wide age range of radiographic appearances in pediatric elbow joints. The ossification center of the trochlea of the humerus typically appears around the age of 9 years. Before the appearance of ossification centers, medial condyle fractures of the humerus may not be evident on radiographic examination, manifesting only as soft tissue swelling or positive fat pad signs around the elbow joint. This lack of characteristic radiographic findings may contribute to underdiagnosis or misdiagnosis, particularly among less experienced clinicians (4–6). Medial condyle fractures of the humerus in young children are often misdiagnosed as medial epicondyle fractures based on clinical manifestations and imaging. However, unlike medial condyle fractures of the humerus, medial epicondyle fractures only involve the extra-articular fracture of the medial epicondyle, while medial condyle fractures of the humerus are intra-articular fractures that may involve the trochlea of the humerus, the medial epicondyle, and part of the metaphyseal region of the humerus, presenting as a mirror-image symmetric injury to lateral condyle fractures. From a Salter-Harris classification perspective, these fractures are categorized as Salter-Harris type IV intra-articular epiphyseal fractures (7). Achieving anatomical reduction is essential, as inadequate reduction or improper management may lead to complications such as nonunion, malunion, osteonecrosis of the epiphysis, or elbow joint deformities (8), which can have irreversible consequences on elbow joint function and psychological well-being.

Therefore, early and accurate diagnosis, timely and appropriate reduction and fixation of fractures, and rational rehabilitation therapy are crucial for prognosis. Currently, the fracture fixation materials used in orthopedic surgeries are primarily made of metallic implants such as stainless steel, titanium, and cobalt-chromium-based alloys. Despite their high mechanical strength and stability, these materials cause long-term stress shielding due to the difference in strength and hardness between them and bone. Additionally, these metallic implants may cause residual pain, irritation, or inflammatory reactions, necessitating their removal in the later stages after fracture healing (9). In the search for the optimal biodegradable biomaterials, magnesium (Mg) possesses some unique properties that make it an attractive material for orthopedic implants. However, the first implants were made from pure magnesium, which underwent rapid degradation, and the results were not as expected. The rapid degradation led to a quick loss of biomechanical properties. Although the modified Mg-based absorbable cannulated screws have slowed down the degradation rate, a large amount of degradation product, especially hydrogen gas, has accumulated in the tissue, forming magnesium hydroxide. This can lead to local acidity buildup, causing inflammation or tissue irritation, which affects the bone healing process (10–12).

On the other hand, PLGA-based absorbable cannulated screws, although having a lower mechanical strength than Mg, are favored due to their more gradual degradation process and good biocompatibility. Additionally, PLGA is more cost-effective than magnesium alloys, and its production process is relatively mature, making it widely used in clinical settings for minor fracture repairs or for areas that require support for lighter loads (13).

This study is a single-center retrospective analysis. Due to limited research on medial condyle fractures of the humerus in children, this study exclusively examined 23 cases of Kilfoyle type II and III medial condyle fractures in children that were electronically registered and treated with open reduction through a medial approach to the elbow joint and internal fixation with absorbable pins at Foshan Hospital of Traditional Chinese Medicine between June 2018 and December 2023. Seven surgeons participated in this study. The treatment outcomes were satisfactory, as described below.

## General information

The inclusion and exclusion criteria are shown in Table 1.

**Table 1:** Inclusion and exclusion criteria.

Inclusion criteria	Exclusion criteria
1. Age ≤14 years old. 2. Parental consent for surgical 3. Confirmation of pediatric Kilfoyle II or III type medial condyle fractures of the humerus through elbow joint ultrasound, MRI, CT or x-rays. treatment.	1. Pathological medial condyle fractures of the humerus. 2. Kilfoyle type I pediatric medial condyle fractures of the humerus. 3. Presence of vascular or nerve injuries or open Kilfoyle II or III type pediatric medial condyle fractures of the humerus. 4. Parental refusal of surgical treatment.

## Surgical procedure

**Figure 1:**
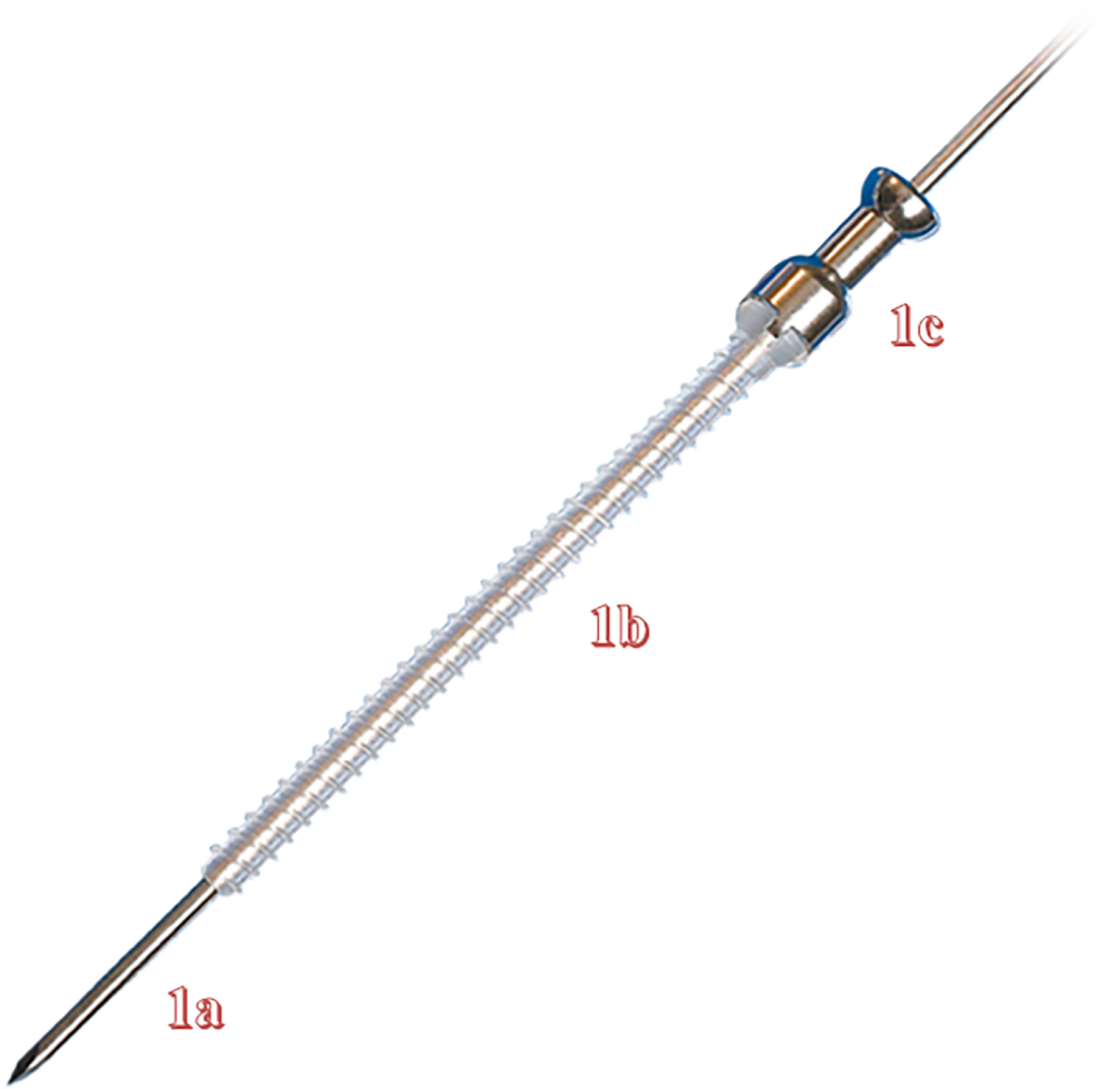
Guidewire **(a)**, absorbable cannulated screw **(b)**, screwdriver **(c)**.

The patients were placed in a supine position and underwent intravenous anesthesia induction followed by endotracheal intubation for general anesthesia. Hemostasis was achieved in the affected limb, and then, through the elbow medial approach, the deep fascia was incised. The ulnar nerve was identified and protected with a film. The fracture ends were fully exposed, blood clots and soft tissues were removed, and the fracture was irrigated and reduced. (If it is a neglected fracture, the fracture surface is freshened and then reduced). A towel clamp was used to fix the bone fragments, and two appropriately sized K-wires were crossed to stabilize the fracture fragments. Confirm the fixation of the K-wires and ensure the alignment of the fracture, adjusting the depth of the K-wires as necessary. Additionally, another K-wire was selected to ensure fixation in a different plane, and its position was confirmed. Once stability was achieved, the length of the first K-wire was measured, removed, and replaced with a guide wire. An absorbable cannulated screw, with a length matching that of the K-wire, was then slowly inserted using a screwdriver in the same direction as the first wire, and then the guide wire was pulled out. If resistance was encountered, the screw insertion direction was checked to ensure alignment with the original wire pathway, and soft tissue entrapment was evaluated. After that, the second absorbable cannulated screw was used in the same way. After removing the third K-wire, the decision to insert the third absorbable cannulated screw was based on the stability of the fracture fragments. The ulnar nerve was re-examined, loosened if compressed, and protruding absorbable cannulated screws were trimmed. The wound was irrigated with saline, and the bone membrane was sutured using absorbable sutures. Ligaments and joint capsules around the joint were repaired to stabilize the elbow joint, followed by layered closure of the incision. In this study, we utilized the fourth-generation BioFix bioabsorbable full-threaded screws produced by Bioretec Finland, which are made of poly (L-lactide-co-glycolide) (PLGA) and have a diameter of 2.0 mm. As shown in Figure 1. All surgeries were performed by a chief physician specializing in pediatric orthopedics with over 20 years of experience, assisted by a physician with more than 5 years of experience. According to the classification by Tang and Giddins, this corresponds to Level 3: Specialist-experienced and Level 4: Specialist-highly experienced (14).

## Observation indicators

After surgery, the affected limb was immobilized with a plaster splint in 90° of flexion and supination. Once sensation and motor function in the affected limb recovered, active fist-gripping exercises were initiated under guidance. The follow-up appointments were scheduled at 1, 4, and 8 weeks post-discharge, followed by regular appointments thereafter. At the 4-week postoperative visit, anteroposterior and lateral x-rays of the elbow joint were obtained to assess the degree of fracture healing. If continuous callus formation across the fracture line was observed, the plaster splint was removed, and active flexion-extension exercises of the elbow joint were initiated, while passive joint exercises were prohibited. During the final follow-up visit, the Broberg-Morrey elbow joint functional scoring system (15) was utilized to assess the treatment efficacy, with a maximum score of 100 points. The assessment was reported by clinicians rather than by patients. Scores ranging from 95 to 100 were considered excellent, 80 to 94 were considered good, 60 to 79 were considered fair, and 0 to 59 were considered poor. Additionally, the carrying angle of the affected side, maximum extension angle, and maximum flexion angle of the elbow joint were measured based on anteroposterior x-rays of the elbow joint taken at the 6-month postoperative visit. To minimize measurement errors, we enlisted the expertise of three pediatric orthopedic surgeons with over 15 years of experience to measure each data point and averaged the results.

## Results

### Clinical data

This group comprised a total of 23 cases, including 15 males and 8 females. Their ages ranged from 5 to 12 years, with a mean age of 9.0 ± 2.4 years. According to the Kilfoyle classification, there were 3 cases of type II and 20 cases of type III fractures. The mechanisms of injury included falls in 5 cases and sports-related injuries in 18 cases. Among the cases, 10 involved the left side and 13 involved the right side. According to the time of fracture injury, there were 4 cases of neglected fractures and 19 cases of fresh fractures. All patients with fresh fractures underwent surgery within one week of injury or within three days of admission. For patients with neglected fractures, surgery was completed within three days of admission. The average time from the initial injury to hospitalization and surgical treatment at our hospital for the 4 patients with neglected fractures was 73 days. Clinical presentations commonly included pain and tenderness on the medial aspect of the elbow joint, accompanied by varying degrees of restricted elbow joint motion.

All patients' basic information is shown in Table 2. All 23 cases were successfully operated on and completed the full follow-up period, ranging from 3 to 12 months, with an average of 7.5 months. The time to fracture healing ranged from 4 to 8 weeks, with an average of 5.5 weeks. Since the ulnar nerve was exposed and protected during the surgery, no symptoms related to ulnar nerve injury were observed postoperatively. None of the patients experienced complications such as nonunion, fixation device fracture, elbow varus deformity, or nerve injury. Since medial condyle fracture of the humerus is an intra-articular fracture involving a wide range and large fracture fragments, no patient was fixed only with one screw, 22 patients were fixed with two screws, and one older patient was fixed with three screws. At the 6-month postoperative follow-up, the efficacy was evaluated according to the Broberg-Morrey elbow joint functional scoring system: 21 cases were rated as excellent, and 2 cases were rated as good. At 6 months postoperatively, the affected side's carrying angle ranged from 5.6° to 15.9°, with an average of 10.1°; the maximum extension angle of the elbow joint ranged from −4.6° to 0°, with an average of −2.1°; and the maximum flexion angle ranged from 122.2° to 135.6°, with an average of 127.3° (Table 3).

**Table 2:** Basic information of 23 patients.

Basic information	Number	Mean
Sex (male/female)	15/8	–
Age (years)	5–12	9.0 ± 2.4
Injured Side (left/right)	10/13	–
Kilfoyle type (II/III)	3/20	–
Fracture type (fresh/neglected)	19/4	–

**Table 3:** Clinal result of 23 patients.

Observation indicators	Number	Mean
Fracture healing time (week)	4–8	5.5
carrying angle	5.6°–15.9°	10.1°
maximum extension angle	−4.6°–0°	−2.1°
maximum flexion angle	122.2°–135.6°	127.3°
Broberg-Morrey scoring	92–99	97.1
Excellent/good	21/2	–

**Figure 2:**
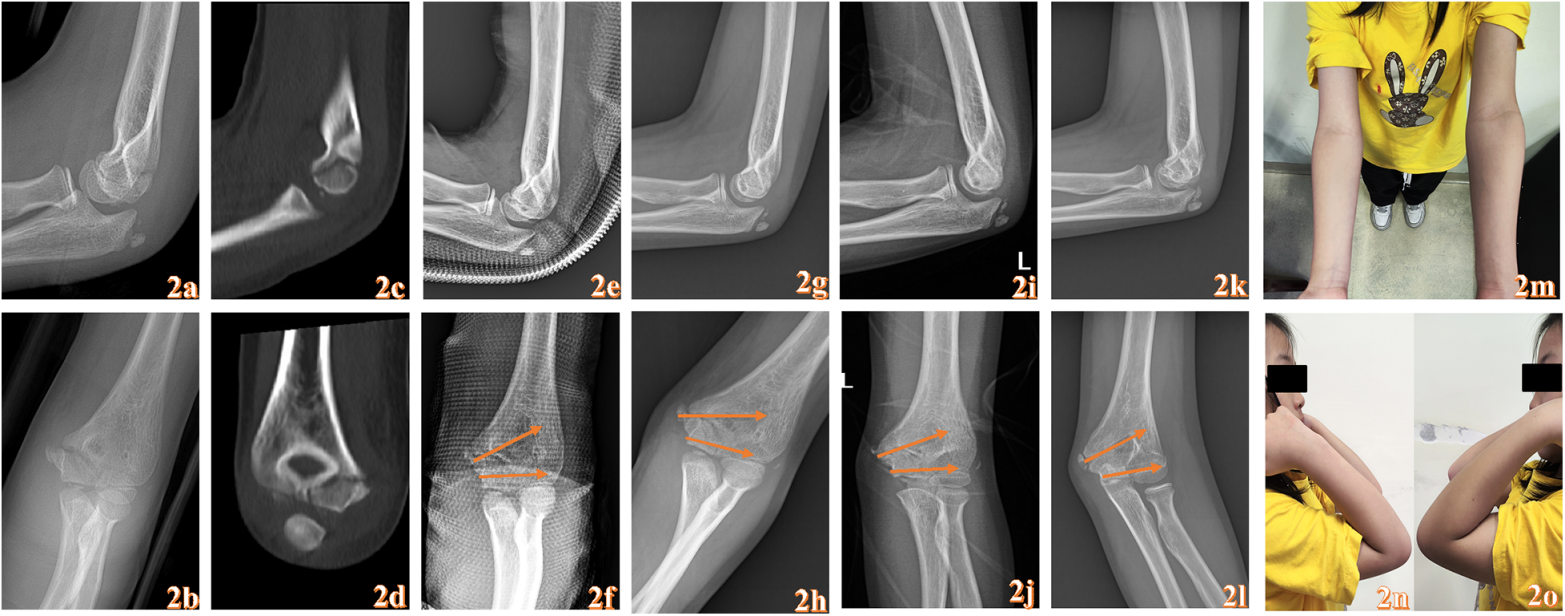
An 8-year-old female child who suffered pain in her left elbow due to a fall while running “pain with functional limitation 3d” admission. Anterolateral x-rays of the elbow joint before surgery **(a,b)**. Preoperative CT **(c,d)**. the anterolateral x-ray after operation **(e,f)**. Postoperative anterolateral x-rays after 1 month **(g,h)**. Postoperative anterolateral x-rays after 3 months **(i,j)**. Postoperative anterolateral x-rays after 6 months **(k,l)**. Appearance photographs taken 6 months postoperatively **(m–o)**. The yellow arrow represents the placement of the absorbable cannulated screws.

Typical Case 1 is shown in Figure 2.

## Discussion

Pediatric medial condyle fractures of the humerus typically occur when a child falls with the elbow extended and supported with force, resulting in direct impact to the medial aspect of the elbow joint or when the elbow joint is extended and subjected to varus stress, causing avulsion fractures (2). The incidence of pediatric medial condyle fractures of the humerus is relatively low, with a higher prevalence among children aged 8–12 years old. The mean age of the children in this study was 9.0 ± 2.4 years, which is consistent with previous literature (16). In recent years, there have been reported cases occurring in children under 6 years old, with some cases even reported in children as young as 2 years old (17, 18). The ossification centers in the trochlea of the humerus typically begin to appear around age 9, with boys exhibiting a delay of 1–2 years compared to girls (19).

In older children and adolescents, medial condyle fractures of the humerus are more easily diagnosed on x-ray due to the visibility of the fracture line and changes in the position of the epiphysis. When combined with the patient's history of injury and positive clinical signs in the elbow region, clinicians can more accurately diagnose the condition. However, for younger children, especially those under 5 years old, the ossification center of the medial condyle is not visible on x-ray, which may result in potential underdiagnosis and misdiagnosis (20). During childhood, ligamentous tissue is significantly stronger than epiphyseal and cartilaginous tissue, making epiphyseal fractures more likely to occur than ligament injuries or joint dislocations. Therefore, in young children with a clear history of elbow injury and obvious positive signs on clinical examination, but no obvious findings on x-rays, attention should be paid to the possibility of a fracture (21).

Firstly, attention should be paid to the presence of a positive fat pad sign (22) x-ray examination of the elbow joint. Stress views and arthrography can be used for further diagnosis, although these methods are invasive and may be challenging for children to cooperate with during the procedure. In recent years, the application of musculoskeletal ultrasound imaging has provided significant assistance in the diagnosis and treatment of pediatric elbow joint fractures (23). Additionally, MRI examination can provide information on the location and displacement of fractures, which is crucial for the diagnosis and treatment of epiphyseal fractures (24, 25).

On the other hand, pediatric medial condyle fractures of the humerus involve the joint surface, trochlear ossification center, epiphyseal plate, and distal humeral metaphysis, which are classified as Salter-Harris type IV intra-articular epiphyseal fractures (7). Once diagnosed with the medial condyle fractures of the humerus with displacement, surgical treatment is recommended to achieve anatomical reduction and stable fixation. Failure to achieve satisfactory reduction and stable fixation can lead to elbow joint dysfunction and limb deformities, resulting in a series of sequelae (1).

Currently, the main treatment methods for pediatric medial condyle fractures of the humerus include closed reduction with splint immobilization, closed reduction with plaster cast immobilization, closed reduction with K-wire fixation, and open reduction with K-wire fixation. Closed reduction with splint immobilization or plaster cast immobilization is suitable for non-displaced Kilfoyle type I fractures or minimally displaced type II fractures. However, simple plaster or splint external fixation may fail to maintain reduction and can lead to fracture second displacement, especially after the swelling subsides. This is shown in Typical case 2. Many researchers believe that Kilfoyle type I and non-displaced type II fractures can be treated non-surgically (3, 26). However, type II fractures involve complete fracture lines and have a high risk of late displacement. Once displacement occurs, treatment becomes challenging. Additionally, most children exhibit increased activity after pain relief, making it difficult to assess fracture reduction on follow-up x-rays, especially in younger children. Moreover, the interference of external fixation devices makes it challenging to evaluate whether there is fracture second displacement while maintaining external fixation. Therefore, we recommend surgical treatment for completely fractured type II medial condyle fractures of the humerus (Figure 2). In our study, all 3 cases of Kilfoyle type II fractures showed good treatment outcomes.

Kilfoyle type III fractures often involve severe soft tissue damage to the joint capsule and surrounding ligaments, accompanied by more severe injuries such as fracture block rotation, posterior dislocation of the elbow joint, or block impingement. Therefore, aggressive surgical treatment is required in these cases. Open reduction with internal fixation surgery generally yields better reduction outcomes, but the choice of internal fixation material can significantly impact the surgical outcome and the incidence of complications. K-wire is commonly used internal fixation materials for pediatric medial condyle fractures of the humerus. Their advantages include simplicity of operation, reliable fixation, the ability to stabilize small fracture fragments, and minimal risk of damaging the ossification center (8). However, there are reports in the literature suggesting that closed reduction with K-wire fixation for pediatric elbow fracture may lead to nerve or vascular injury, while open reduction with K-wire fixation may result in complications such as pin tract infection or osteomyelitis (27). Additionally, a second surgery is often necessary to remove the K-wires after fracture healing, which adds to the child's discomfort.

With the gradual improvement of minimally invasive surgical techniques and increasing expectations from families of affected children, reducing or even avoiding postoperative complications of internal fixation for pediatric medial condyle fractures of the humerus while minimizing surgical trauma has become a challenge for clinical physicians. Absorbable cannulated screws, composed of materials such as polypropylene and polyethylene, are non-toxic and biocompatible and do not elicit any immune response. They are absorbed by the body as the patient recovers, eventually hydrolyzing into water and carbon dioxide (28). Absorbable cannulated screws have minimal stress shielding effects, comparable initial strength to cancellous bone, and undergo rapid expansion and shortening upon implantation into the body, thereby exerting automatic compression on the fracture site. As the absorbable cannulated screws degrade and are absorbed, stress gradually transfers to the fracture site, promoting fracture healing, with strength sustained for up to six months to two years.

Compared to K-wire fixation, choosing absorbable cannulated screws fixation for pediatric medial condyle fractures of the humerus can provide better biomechanical stability and avoid the need for a second surgery. The application of absorbable cannulated screws has been widely recognized by clinicians and patients alike. Opting for absorbable cannulated screws fixation can spare children the pain of a second surgery to remove K-wire, reduce treatment time and costs, alleviate patient discomfort, and facilitate fracture healing. Certainly, some authors have reported issues such as local rejection reactions and degradation absorption time with the use of absorbable screws as internal fixation devices. These issues certainly merit attention. However, in this study, we did not observe problems caused by the aforementioned situations, which may be partially related to the relatively small sample size and insufficient follow-up duration (29).

## Treatment experience and shortcomings

As discussed above, the treatment of pediatric medial condyle fractures of the humerus, once diagnosed correctly, is not particularly challenging, and satisfactory therapeutic outcomes can often be achieved with anatomical reduction and internal fixation. In this study, 23 cases of Kilfoyle type II and III medial condyle fractures of the humerus were treated using an approach involving open reduction and internal fixation with absorbable cannulated screws via the medial approach of the elbow joint. Ultimately, all patients achieved bony union, and satisfactory elbow joint function was observed in the six months following surgery, with no occurrences of cubitus varus or valgus deformities, or internal fixation failure. Severe medial condyle fractures of the humerus often involve ulnar nerve injury, or even entrapment, making it difficult to achieve satisfactory reduction. However, the medial approach to the elbow joint provides a clear surgical field, allowing direct exposure of the fracture site and facilitating precise anatomical reduction. Additionally, this approach facilitates exploration and exposure of the ulnar nerve, reducing the risk of damage to the surrounding soft tissues and minimizing complications such as avascular necrosis and nonunion, thereby reducing iatrogenic injury. The use of absorbable cannulated screws as internal fixation material offers excellent biocompatibility, obviating the need for secondary surgery, thereby reducing patient discomfort and promoting postoperative recovery. Moreover, it helps to prevent pin tract infections and enables early initiation of elbow function exercises, thereby improving elbow joint function.

This study also has some shortcomings. Firstly, there is a lack of a control group in this study. Due to the low incidence rate of the disease, the number of patients is relatively small, and it is not feasible to collect a sufficient number of cases for a randomized controlled trial within a short period. Secondly, the growth disturbance or elbow joint deformity caused by osteonecrosis after distal humeral epiphyseal fracture requires at least 2–4 years of follow-up (30). The follow-up period in this study was relatively short. Furthermore, the price of cannulated screw is relatively higher compared to traditional Kirschner wires and cannulated screws, approximately twice as much. This is also an important reason why it is difficult to conduct this research on a large scale clinically. The next step will be to continue follow-up and, as much as possible, confirm the effectiveness and safety of absorbable cannulated screws treatment for medial condyle fractures of humerus through prospective randomized controlled trials.

## Conclusion

This study retrospectively analyzed 23 cases of Kilfoyle II and III type medial condyle fractures of humerus in children treated with open reduction and internal fixation using absorbable cannulated screws via a medial approach at the Pediatric Orthopedics Department of Foshan Hospital of Traditional Chinese Medicine from June 2018 to December 2023. The results indicated that the medial approach provided clear visualization of the fracture fragments, facilitated exploration of the ulnar nerve, aided in fracture reduction, and resulted in inconspicuous incisions postoperatively. The use of absorbable cannulated screws demonstrated good biocompatibility and reliable fixation, avoiding the need for secondary surgery postoperatively, thereby effectively reducing treatment time and costs while alleviating patient discomfort. In summary, open reduction and internal fixation with absorbable cannulated screws via a medial approach showed satisfactory clinical efficacy in treating Kilfoyle II and III type medial condyle fractures of humerus in children, making it a favorable method for the treatment of such fractures in clinical practice, warranting further promotion and application.
